# Apoptosis: A Key Process That *Trypanosoma cruzi* Modulates as a Strategy to Perpetuate Infection

**DOI:** 10.1155/japr/2093615

**Published:** 2025-07-07

**Authors:** Diego Maurizio Coria-Paredes, Arturo A. Wilkins-Rodríguez, Laila Gutiérrez-Kobeh

**Affiliations:** ^1^Research Unit UNAM-INC, Research Division, Faculty of Medicine, National Autonomous University of Mexico-National Institute of Cardiology “Ignacio Chávez”, Mexico City, Mexico; ^2^Doctoral Program in Biological Sciences (Posgrado en Ciencias Biológicas), Faculty of Medicine, Postgraduate Unit, University City, Mexico City, Mexico

**Keywords:** apoptosis, cardiomyocytes, chagasic cardiomyopathy, macrophages, signaling pathways, T lymphocytes, *Trypanosoma cruzi*

## Abstract

Apoptosis is a crucial host defense mechanism because it can trigger the immune response and get rid of infected cells. These important goals, among others, are achieved through a fine-tuned process that culminates in a quiet form of cell death. Apoptosis represents a hindrance for intracellular microorganisms that live inside cells. Nevertheless, they have developed strategies that allow them to survive in diverse microhabitats inside their hosts, avoiding the immune response defense mechanisms. A good example of this is the intracellular parasite *Trypanosoma cruzi*, which, thanks to various adaptations, manages to go through its life cycle passing through the digestive tract of hemiptera and mammalian blood to reach its destination: the cytoplasm of the cells it infects. *T. cruzi* causes Chagas disease or American trypanosomiasis that constitutes a major health issue worldwide with 6–7 million people currently infected and approximately 10,000 annual deaths. Infection with *T. cruzi* can cause an extensive range of disease going from acute to chronic forms such as the chronic chagasic cardiomyopathy. In the transition from one form to another, many factors are implicated where the host immune response and *T. cruzi* genetic diversity stand out. Being an intracellular parasite, *T. cruzi* must evade the host's defense mechanisms to successfully establish an infection. Apoptosis represents one of these mechanisms, and *T. cruzi* has developed several strategies to inhibit or induce apoptosis depending on the cell context. Interestingly, in addition to the ability of *T. cruzi* to modulate host apoptosis, it can experience an apoptosis-like cell death. In this review, we describe apoptosis and its main paths of induction as well as basic aspects of the parasite and Chagas disease and examine how the regulation of apoptosis influences infection by this protozoan.

## 1. Introduction

Apoptosis is a delicately tuned mechanism in which cells completely dismantle. It plays a key role in processes such as embryogenesis, tissue reorganization, the discarding of harmed or useless cells, and immune defense. This last process represents an obstacle for pathogens that need to modulate apoptosis to persist in the host. This modulation is very well exemplified by the intracellular parasite *Trypanosoma cruzi* (Kinetoplastidae: Trypanosomatidae), which is the etiologic agent of the zoonosis American trypanosomiasis or Chagas disease (CD). This pathology was initially described in 1909 by the Brazilian physician Carlos Chagas, and since then, more than 150 wild and domestic mammals have been found to act as reservoirs [1, 2]. The main pathway of infection to humans is through insect vectors of the Triatominae subfamily [3]. Infection with this parasite can have a long duration and, if left untreated, patients can develop dangerous pathologic situations as exemplified by the chagasic cardiomyopathy (CC), which can have an acute manifestation or appear after decades of being latent [4]. In this last scenario, *T. cruzi* needs to survive inside host cells for long periods and thus is imperative for the parasite to resist host defense mechanisms. Thus, *T. cruzi* has developed a wide variety of mechanisms to tip host immune response on its behalf [5–11]. One of the key strategies employed by *T. cruzi* to maintain long-term infection in vertebrate hosts is the induction of T and B cell apoptosis, which enables immune evasion and modulates the host's immune response [12–16].

### 1.1. Apoptosis

The term apoptosis comes from two Greek roots, apo and ptosis, meaning to fall into pieces, and comprises a genetically ordered process that conducts to the destruction of the cell [17]. Cells that experience this type of cell death traverse through different morphological and biochemical changes that truly resemble the idea of falling into pieces. Some of these changes include cell shrinkage and cytoskeletal collapse, disassembly of the nuclear envelope, and chromatin condensation and fragmentation. Another affected organelle is the mitochondria, where pores couple both mitochondrial membranes and release luminal mitochondrial components in between the intermembrane space causing loss of membrane potential. The cell membrane also suffers chemical changes such as the expression of phosphatidylserine (PS) in the external leaflet, which is promptly recognized by cells of the immune system, such as macrophages, and the early elimination of cellular debris prevents inflammation [18]. It also emits protrusions and breaks into pieces surrounded by the cell membrane called apoptotic bodies. These features have been used to quantitatively evaluate apoptosis [19, 20]. Nevertheless, the Committee for Nomenclature of Cell Death suggests that quantifying biochemical parameters is the clearest approach to defining the different types of cell death [20]. The central role of apoptosis in embryogenesis, tissue reconstruction, and the discarding of deteriorated or nonfunctional cells makes it a vital step in the development of metazoans [21]. On the other hand, for intracellular pathogens, host cell apoptosis represents a two-way choice. If delayed or inhibited, it represents an opportunity to survive for longer in host cells, but if induced, it may be favorable for the spreading of infection, or it could favor the control of the infection by preventing the invading parasite from completing its life cycle [22]. It has been shown that during chronic infections by intracellular pathogens, several antigens or secreted factors are able to trigger apoptosis [23, 24].

### 1.2. Apoptosis Activation Phases

For apoptosis to occur, it must transit through an initiation and an execution phase. During the first one, genes and signaling pathways are activated, while the second one depends on a series of proteases called caspases, which possess cysteine residues in their active sites. They cleave their substrates at sites following aspartic acid residues and are functionally categorized into initiators (Caspases 8, 9, and 10) and effectors (Caspases 3, 6, and 7) [25]. Initiator caspases have multiple targets, which include executioner caspases. The activity of both types of caspases is responsible for the morphological changes typical of apoptosis [26], ultimately leading to the disassembly of the cell. At the same time, the initiation phase of apoptosis can be triggered in several ways: (1) the extrinsic pathway, activated by receptors; (2) the perforin/granzyme pathway; and (3) the intrinsic pathway. The extrinsic pathway is triggered when soluble ligands bind to receptors, primarily from the TNF family, which are characterized by the presence of death domains (DDs). The perforin/granzyme pathway involves the action of molecules found in cytotoxic cells, such as natural killer (NK) cells and cytotoxic T lymphocytes. The intrinsic or mitochondrial pathway is primarily triggered by cellular stress, which can result from various events, including DNA damage, nutrient deprivation, and the accumulation of misfolded proteins, among others. Interestingly, this division of the induction pathways of apoptosis is only for didactic purposes since the different pathways can activate each other and are not mutually exclusive. Thus, the execution of the cell begins with the first caspases after activation and culminates with the hallmark features of apoptosis that include DNA fragmentation, degradation of nuclear and cytoskeletal proteins, exposure of ligands recognized by phagocytic cell receptors, formation of apoptotic bodies, and ultimately, phagocytosis by neighboring cells [17].

### 1.3. The Extrinsic Pathway

**Figure 1:**
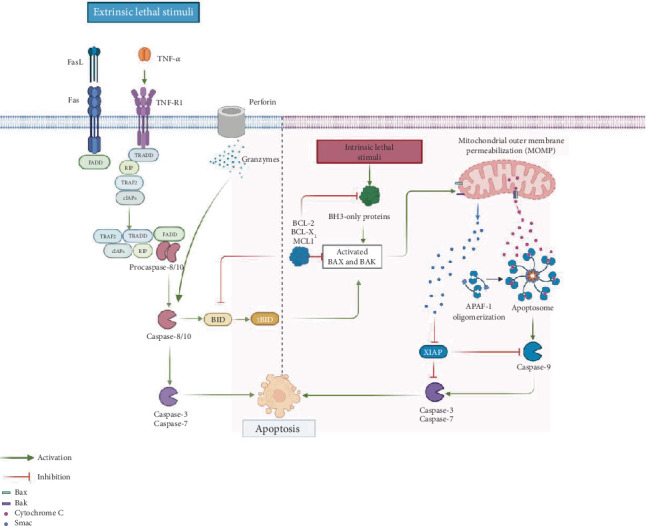
Extrinsic and intrinsic pathways of apoptosis. Apoptosis via the extrinsic pathway is initiated through the activation of death receptors such as TNFR and FAS, which belong to the TNF receptor family. Upon binding to their respective ligands, these receptors recruit adaptor proteins like TRADD and FADD. These adaptor proteins assemble into multiprotein complexes with the intracellular domains of the receptors and other molecules, ultimately leading to the activation of initiator caspases, primarily Caspase 8. Caspase 8 then activates executioner caspases, mainly Caspase 3, resulting in cell apoptosis. The intrinsic pathway, on the other hand, is triggered by mitochondrial outer membrane permeabilization (MOMP), which leads to the release of proapoptotic factors. One key factor is cytochrome c, which, together with APAF1, forms the apoptosome. This complex enables the autoactivation of Caspase 9 and subsequently activates Caspases 3 and 7. Notably, the cleavage of BID into tBID serves as a positive feedback mechanism, linking the extrinsic and intrinsic pathways by promoting mitochondrial pathway activation.

As the name implies, the binding of soluble ligands to cell surface receptors belonging to the tumor necrosis factor receptor (TNFR) superfamily initiates this pathway. These receptors are characterized by the presence of DDs, which are intracellular regions of approximately 80 amino acids. Members of this family include Fas receptor and its ligand (FasL), tumor necrosis factor *α* (TNF-*α*) and its receptor TNFR1, Apo3L/DR3, and TRAIL and its receptors (TRAILR), as well as netrin receptors (UNC5A-D). The best researched members of the family are FasR and TNFR, from which many aspects of the extrinsic pathway have been unveiled. Both receptors are found in the cell membrane as labile homotrimers that, upon binding with their respective ligands, are stabilized and suffer conformational changes in the DD. This recruits cytosolic factors such as FADD (DD union protein associated to FAS) or TRADD (protein with DD associated to TNFR). Then, the initiator Procaspases 8 and 10, mainly 8, are autocatalytically activated through their association with the death effector domain (DED) of FADD. At the same time, these active caspases activate the Executioner Caspases 3, 6, and 7, most commonly 3, and are responsible for the turnover of the cell [27]. If TNFR is the receptor engaged, the story is more complex since several molecules are involved. TRADD recruits RIP and TRAF to form Complex I [28] but also apoptosis inhibitory proteins (c-IAP) 1 and 2 can be recruited to TRAF2 [29]. RIP kinase is ubiquitinated by c-IAP 1/2 and inhibits apoptosis and promotes cell survival through the activation of NF-*κ*B [30]. Contrarily, the inhibition of c-IAP 1/2 releases TRADD-RIP (Complex II) from TNFR by interfering with RIP ubiquitination. Then, Complex II recruits FADD in the cytoplasm that binds and activates Procaspase 8 and subsequently results in the activation of executioner caspases [17, 28, 31] (Figure 1).

### 1.4. The Perforin/Granzyme Pathway

The key initiators of this pathway are perforin and granzymes, which are stored in the granules of cytotoxic immune cells such as NK cells and cytotoxic T lymphocytes. These cells play a crucial role in eliminating abnormal cells, including tumor cells or those infected with intracellular pathogens. Upon recognizing a target cell, the cytotoxic cell releases its granule contents—perforin forms pores in the target cell membrane, allowing the entry of serine proteases, specifically Granzymes A and B. Notably, both granzymes can induce apoptosis. Granzyme A triggers DNA fragmentation in a caspase-independent manner by degrading SET, a nucleosome-associated protein complex that normally inhibits the NM23-H1 gene, thereby initiating the production of the DNase NM23-H1. Granzyme B, on the other hand, directly cleaves and activates Procaspase 3, can also activate Procaspase 10, and initiates the caspase cascade leading to the activation of executioner caspases (3, 6, and 7). Additionally, Granzyme B can degrade the inhibitor of caspase-activated DNase (ICAD), further promoting DNA fragmentation [32].

### 1.5. The Intrinsic Pathway

This pathway has been subdivided into two main pathways: the mitochondria-based pathway and a mitochondria-independent endoplasmic reticulum (ER) pathway. The mitochondrial pathway of apoptosis is primarily triggered by cellular stress, which can arise from various insults such as DNA damage, oxidative stress, radiation, hypoxia, or nutrient deprivation. In contrast, an increase in protein secretion or disturbances in protein folding can lead to ER stress, marked by the accumulation of unfolded or misfolded proteins within the ER lumen. These disruptions in ER function are regulated by a complex network of signaling pathways collectively known as the unfolded protein response (UPR), which is activated by ER-resident transmembrane protein sensors: protein kinase R (PKR)-like endoplasmic reticulum kinase (PERK), inositol-requiring enzyme 1 alpha (IRE1*α*), and activating transcription factor 6 (ATF6) [33]. Irrespective of the cause of cellular stress, the initiation of the intrinsic pathway drifts in the activation of the proapoptotic proteins Bak and Bax that polymerize and form pores in the mitochondrial outer membrane and cause its permeabilization (MOMP). This process represents a point of no return in apoptosis because it unchains multiple lethal reactions such as the impairment in ATP synthesis, active transport, and respiratory chain. As a result of the loss of mitochondrial membrane potential, various toxic proteins—such as cytochrome c, apoptosis-inducing factor (AIF), endonuclease G (EndoG), direct inhibitors of apoptosis protein (IAP)-binding protein with low pI (DIABLO or SMAC), and high temperature requirement protein A2 (HTRA2)—are released from the mitochondrial intermembrane to the cytoplasmic space. The liberation of cytochrome C permits the formation of the apoptosome through its union to and activation of the adaptor protein APAF1. The apoptosome, a multiprotein complex, recruits Procaspase 9, which is activated by autoproteolysis, and for its activation, it is necessary to neutralize procaspase-associated IAPs through the release of SMAC/DIABLO to the cytoplasm. Thus, the apoptosome represents a decisive pivot in the induction of apoptosis [34]. The apoptotic suppression by IAPs requires their direct association with caspases and procaspases (primarily Caspase 3 and 7) and the modulation of and by the transcription factor NF-*κ*B [35]. Finally, the initiation of the executioner phase of apoptosis takes place after Procaspase 3 is activated and triggers cellular changes and downstream effector caspases that can process at least 1000 proteins [36]. Alternately, apoptosis can also be initiated independently of caspases. For example, DNA can be fragmented in segments of 50–300 kb by AIF and EndoG when they translocate to the nucleus. Additionally, the antiapoptotic functions of IAPs are inhibited by SMAC/DIABLO and HTRA2, and the serine protease activity of HTRA2 can contribute to the executioner phase of apoptosis independently of caspases [16, 34, 37].

As previously noted, the various apoptosis-inducing pathways are interconnected. Caspase 8 serves as a link between the extrinsic and intrinsic pathways by cleaving the proapoptotic protein Bid into its truncated form (tBid), which promotes the release of apoptogenic proteins such as cytochrome c, thereby triggering the intrinsic apoptotic pathway [17, 28, 31]. Notably, Bid can also be cleaved by Granzyme B, establishing a connection between the granzyme-mediated pathway and the intrinsic pathway [17] (Figure 1).

## 2. Modulation of Apoptosis by Intracellular Microorganisms

Host survival is crucial for intracellular microorganisms because they have a strong dependency on their host. Once inside the cell, pathogens must evade host defense mechanisms, one of which is apoptosis. The modulation of this defense mechanism is crucial for their subsistence. Thus, during evolution, many strategies have been selected, whether to induce or inhibit apoptosis, that permit microorganisms to survive in the host. The induction of apoptosis by intracellular pathogens prevents their killing by antimicrobial effector molecules and permits their dissemination and invasion of other cells. On the other hand, other microorganisms successfully colonize their host cell thanks to their ability to block apoptosis and thus safeguard reproductive shelters and colonize new cells.

In the case of viruses, they are contained by the host through various mechanisms, including Type I interferons release, neutralization by antibodies, and cytotoxic T cell (CTL) activation [38]. One recurrent approach employed by viruses is the induction of apoptosis of T and B cells, as has been demonstrated in HIV-1-infected individuals, where a correlation has been found between viral RNA levels in plasma and apoptosis of CD4^+^ T lymphocytes and CD19^+^ B cells [39].

To induce or inhibit apoptosis, intracellular microorganisms take advantage of the key molecules that participate in the activation of apoptosis. Among these, Fas/FasL, due to their crucial role in the initiation of the extrinsic pathway, are one of the primary objectives of intracellular pathogens to obstruct apoptosis. For instance, the intracellular facultative fungus, *Cryptococcus neoformans*, is the major encapsulated fungal pathogen of humans. It possesses a capsule composed of galactoxylomannan (GXM) and glucuronoxylomannan (GalXM) that has been shown to induce FasL-dependent macrophage apoptosis. In addition, both components have been shown to be immunosuppressive [40].

Among intracellular pathogens, bacteria have been extensively studied in their ability to inhibit apoptosis. It has been shown that they display a wide array of strategies to control this type of cell death. They can interfere with the death receptor-mediated pathway; for example, the plasminogen activator protease, Pla, secreted by *Yersinia pestis*, can block the initiation of apoptosis through the ligation of FasL and the activation of Caspase 3/7 [41]. *Chlamydia trachomatis* also interferes with the extrinsic pathway, blocking TNF-induced apoptosis [42]. Additionally, some bacteria have been shown to stimulate or restrain apoptosis by targeting NF-*κ*B through Type III or Type IV secretion systems [43].

Intracellular parasites have also developed different mechanisms to modulate apoptosis. A very interesting example is *Leishmania.* Particularly, the infection of macrophages with *Leishmania amazonensis* can have different outcomes depending on the interaction with necrotic or apoptotic neutrophils. Interestingly, when *Leishmania*-infected macrophages phagocytose apoptotic neutrophils, there is an increase in parasite loads through the participation of TGF-*β*1 and PGE2. Contrarily, the ingestion of necrotic neutrophils by infected macrophages provokes the destruction of *L. amazonensis* [44].

Another intracellular parasite that displays multiple strategies to modulate apoptosis and is the focus of this review is *T. cruzi*.

### 2.1. The Parasite

#### 2.1.1. *T. cruzi* Life Cycle

This intracellular parasite undergoes several developmental stages that alternate between the insect vector—where epimastigotes and metacyclic trypomastigotes (MTs) are found—and the vertebrate host, which harbors blood trypomastigotes and amastigotes [45]. The life cycle of *T. cruzi* begins when a triatomine insect feeds on the blood of an infected mammal, ingesting blood trypomastigotes [2]. These transform into epimastigotes in the vector's midgut, where they replicate and subsequently migrate to the rectum, differentiating into MTs. During a subsequent blood meal, the vector releases MTs in its feces. The parasites then enter the host through the bite wound—facilitated by rubbing or scratching—or through breaks in the skin or mucous membranes [3, 45, 46]. Once inside, the parasites invade nearby macrophages and connective tissue cells. This invasion is mediated by various surface glycoproteins, including gp82, gp83, gp85, and Tc-85, as well as glycoinositolphospholipids (GIPLs) and extracellular and membrane bound galectin 3 (Gal-3) implicated in the anchoring/penetration process [47–51]. Interestingly, some of the molecules involved in the invasion process are differently expressed between MT and tissue cultured-derived trypomastigotes (TCT). One example is gp82 that is highly expressed in MT, triggers a signaling cascade upon binding to its receptor, and promotes the invasion of human epithelial cells in vitro. On the other hand, TCT, the in vitro counterpart of blood trypomastigotes, express gp85 that apparently does not trigger a signaling cascade but interacts with certain enzymes to elicit its effect [52, 53]. Once inside, parasites differentiate into small round-shaped amastigotes that need to dodge host defense mechanisms, one very important being the microbicidal action of phagolysosomes. For this purpose, *T. cruzi* parasites employ a transialidase (TS) and a low pH-dependent pore-forming protein that enables them to escape from the parasitophorous vacuole (PV) into the host cell cytoplasm, where they proliferate through binary fission. Subsequently, the amastigotes elongate, regenerate their long flagella, and differentiate into nonreplicative trypomastigotes, which then spread via the lymphatic system and bloodstream to various tissues [5, 54–57]. Although *T. cruzi* can infect virtually any nucleated cell, it shows a preference for invading macrophages [58], as well as nervous tissue, muscle cells (including cardiac, smooth, and skeletal muscle), and epithelial cells [59]. The life cycle is completed when a triatomine vector ingests blood containing *T. cruzi* trypomastigotes [2, 45].

### 2.2. The Disease

CD is widely distributed in Latin America, where it is present in 21 countries, from Mexico to the south of Argentina and Chile. It is prevalent in rural areas of extreme poverty, where vectors are present that favor transmission and is considered an unattended tropical disease by the World Health Organization (WHO) [60]. Nevertheless, this epidemiological landscape has suffered significant changes during the last three decades. The implementation of vector control strategies—such as the use of insecticides and improvements in housing—has significantly contributed to reducing the number of *T. cruzi* infections [61]. As a result, the estimated number of infected individuals has declined markedly, from approximately 30 million in 1990 to 6–7 million today. This decline has been accompanied by reductions in both incidence and mortality. Reported cases fell from 700,000 in 1990 to 30,000 in 2018, while deaths decreased from 45,000 to 12,000 during the same period [62, 63]. Despite this progress, CD remains a significant public health concern. The use of insecticides has indeed helped to control the presence of vectors, but the existence of other transmission routes has impeded a major decrease in the number of infections. Other forms of transmission include the congenital pathway, blood transfusions, organ transplants, or consumption of food contaminated with triatomine feces. Another important factor that has inhibited a major decrease in CD cases has been the dissemination to urban zones and nonendemic regions due to the migrations of asymptomatic individuals who are in the chronic phase of the disease and thus unaware of it. This has increased the number of cases in countries such as the United States, where recent estimations suggest that approximately 300,000 people could be infected with *T. cruzi* [64]. Only in Los Angeles County, a study revealed a seroprevalence of 5.2% among Latin American immigrants who also suffered cardiac abnormalities [65]. Other countries that have experienced an increase in CD cases are Japan, Australia, Spain, Italy, the United Kingdom, and other European countries where CD is considered an emergent disease [66, 67]. Among these, Spain is the most affected country, where estimations indicate that there may be 50,000 infected individuals, mainly from Latin American origin [68]. In Latin America, the countries with more cases reported are Argentina (1,535,235), Brazil (1,156,821), and Mexico (876,458) followed by Bolivia (607,186), which is the country with the highest prevalence estimated between 6.8% and 18% of the population being seropositive for *T. cruzi* [69].

### 2.3. Phases of CD

In humans, CD progresses through an acute phase followed by a chronic phase, which can be either symptomatic or asymptomatic [70]. The acute phase typically develops 1–2 weeks after parasite inoculation, lasts between 4 and 8 weeks, and is characterized by high blood parasitemia. Interestingly, most acute infections are rarely diagnosed (estimated at less than 1%–5%) when acquired via vector transmission. This is likely because patients exhibit mild and nonspecific symptoms, such as fever, general malaise, and hepatosplenomegaly, which are often misattributed to other conditions. However, in some cases, lesions at the parasite inoculation sites can be observed, such as a cutaneous nodule (inoculation chagoma) or unilateral bipalpebral edema (Romaña's sign) [70, 71]. Fewer than 1% of cases develop encephalitis or myocarditis, which can be fatal, especially in children and the elderly [72]. In contrast, myocarditis tends to be more severe and carries a higher mortality risk when the infection is acquired orally, through the consumption of contaminated food, or in immunocompromised individuals [73]. This may be due to higher parasite loads, increased permeability of the upper mucosa to the parasite, and heightened infectivity of trypomastigotes exposed to gastric acid [74, 75].

### 2.4. Host Defense Against *T. cruzi*

The initial high blood parasitemia that characterizes the acute phase is restrained by the host innate immune response represented mostly by the action of proinflammatory cytokines (e.g., IL-12, TNF-*α*, and IFN-*γ*), reactive oxygen and nitrogen species produced predominantly by macrophages, and perforins secreted by NK cells. In mice, the innate immune response controls blood parasitemia up to Day 13 after infection. After 14 days, the adaptive immune response takes control. In humans, innate and adaptive immune responses also play a part in this process, as patent blood parasitemia lasts much longer [76].

The innate immune response to *T. cruzi* is followed by an adaptive immune response, characterized by the polyclonal activation of CD4^+^, CD8^+^ T cells, and B lymphocytes. These cells work together to effectively eliminate parasites from most tissues. However, the persistence of parasites, particularly in cardiac and smooth muscle tissues, marks the onset of the chronic phase [77–81]. This phase may present with digestive or cardiac symptoms in 30%–40% of infected individuals, typically occurring 10–30 years after the acute phase, while the remaining individuals remain in an indeterminate chronic phase throughout their lives [4, 62, 82]. The onset of CC symptoms may occur sooner than previously thought. Recent studies conducted in children and adolescents from endemic regions of Mexico suggest that CC symptoms can appear just a few months after infection [83, 84].

### 2.5. CC

The most important clinical manifestation of CD is the CC due to its high morbimortality in endemic zones [85, 86]. It is characterized by a complex pathogeny of which many aspects are still under investigation. At present, it is known that the damage to the cardiac tissue is progressive and that the presence of the parasite causes chronic inflammation, myocytolysis, and fibrosis that affects all the heart layers [87]. The most important lesions are localized in the myocardium, which favors heart failure, arrhythmias, and thrombus. The excitation-conductive system also results affected, provoking total and partial blockages of any of the branches of the bundle of His (primarily the right branch) and, in some cases, complete blockages of the atrioventricular (AV) node are affected [88, 89]. The role of the parasite as a significant cause of heart damage in CD has been debated for many years. The controversy stems from the fact that the number of parasites found in the heart tissue of patients with chronic CC is minimal. This low parasite presence, combined with the presence of immunoglobulins that bind to muscarinic and *β*-1 adrenergic receptors on cardiomyocyte surfaces, and the detection of cytotoxic T lymphocytes targeting myocardial fibers, led to the suggestion of an autoimmune etiology [90–94]. However, this theory has evolved over the past three decades due to research conducted in animal models of *T. cruzi* infection and in humans with CD. It is now understood that there is a correlation between the quantity of *T. cruzi* antigens and the inflammatory infiltrate, primarily composed of CD8^+^ and CD4^+^ T lymphocytes and macrophages with a Th1 cytokine profile (TNF-*α*, IFN-*γ*, IL-1, and IL-2), which targets the parasites present in the tissue [95–98]. This Th1 response, typical of many intracellular infections, initially protects the host but becomes tissue-damaging as it progresses, contributing to diffuse myocarditis. Over time, myocarditis can lead to myocytolysis and reparative fibrosis, with interstitial collagen deposition that directly correlates with cardiac dilation in the atria and ventricles, particularly the left ventricle [89, 99], and impaired systolic function. A pathognomonic trait of CC is an apical cardiac aneurism that is observed in advanced stages [87]. Also, studies carried out in patients and postmortem analyses have revealed intracavitary thrombi accompanied by infarcts in several organs such as lungs, kidneys, and brain [100–102].

In addition to the cardiac manifestations, CD can affect the digestive tract where the esophagus and the colon are the most compromised organs. The main anatomic and motor alterations result from chronic inflammatory lesions, focal myositis, fibrosis, and intramural neuron damage [103]. The alterations in esophageal motility can cause several symptoms such as dysphagia, odynophagia, epigastric or retrosternal pain, coughing, and regurgitation. During the evolution of the disease, the esophagus suffers denervation, which turns it incapable of transporting the bolus and retains it at the level of the cardias. This can cause undernourishment, weight loss, and pneumonitis due to repetitive aspiration [104]. Conversely, patients with affectation of the colon, megacolon, go through prolonged constipation that can lead to fecaloma, abdominal distension, and intestinal obstruction. They are treated with laxatives, but still, they worsen and may develop twisted bowel (volvulus) [103, 105, 106].

The development of the two main manifestations of the chronic form of CD has been attributed to different factors where the immune response of the host and the genotype of the infecting *T. cruzi* play a leading role. Some aspects of the role of the host immune response in controlling CD have already been pointed out. In relation to the genetic variability of *T. cruzi*, seven discrete typification units (DTU TcI-VI and TcBat) have been described according to their genetic differences, geographical distribution, association with reservoirs and vectors, pathogenicity, clinical characteristics, and response to the treatment, and they have been accepted by international consensus [107]. Nevertheless, a correlation of a Tc with a particular clinical presentation has not been fully established, although a predominance of one form or another has been observed in some regions. For example, the gastrointestinal form of CD is more frequent in South America than it is in the rest of the continent [3, 108, 109].

Currently, the factors underlying the progression from the indeterminate to the determinate chronic phase of CD remain incompletely understood. As with other infectious diseases, both host and parasite-related factors play a critical role in determining the prognosis of CD.

### 2.6. Apoptosis Modulation by *T. cruzi*

*T. cruzi* is an intracellular parasite that needs to colonize host cells to survive because it has a strong dependency on nucleotide and fatty acid/glucose metabolisms and cellular energy [110]. But, once inside, it needs to protect itself from being eliminated. Thus, *T. cruzi* has developed many exquisite strategies to surpass host defense mechanisms. One of these mechanisms is apoptosis, whose fine modulation is critical for *T. cruzi* to survive in the host, for which it has developed different schemes to both induce or inhibit apoptosis depending on the circumstances.

### 2.7. Apoptosis Induction by *T. cruzi*

As previously stated, the control of *T. cruzi* parasitemia depends largely on a robust Th1 response exerted by CD4^+^ T and CD8^+^ lymphocytes through the production of IFN-*γ*. This cytokine, together with TNF-*α* or LPS, induces the expression of the inducible nitric oxide synthase (iNOS or NOS2) and concomitantly the activation of macrophages through the classical pathway (M1) [111], where the production of nitric oxide (NO) is crucial for the elimination of *T. cruzi* [112]. Hence, one of the most important mechanisms employed by intracellular parasites to enhance their survival and replication is the inhibition of NO produced by macrophages. Interestingly, several intracellular parasites have been shown to deploy an apoptotic feature: the exposure of PS, a process that correlates with the inhibition of NO [113, 114]. This strategy has been demonstrated during the infection of IFN-*γ*+LPS-activated macrophages with *T. cruzi* trypomastigotes, where parasites expose PS in their membrane. The interaction of PS with phosphatidylserine receptor (PSR) leads to the secretion of IL-10, TGF-*β*, and PGE2 by the cells that phagocytose apoptotic cells. This signaling pathway is one of the primary anti-inflammatory routes, avoiding apoptosis-induced inflammation [115]. TGF-*β* plays a crucial role in the control of inflammation, managing the secretion of thromboxanes, leukotrienes, and NO [116].

**Figure 2:**
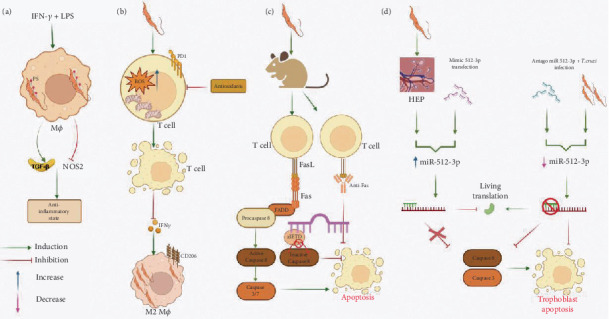
Induction of apoptosis by *Trypanosoma cruzi.* (a) When *T. cruzi* trypomastigotes infect IFN-*γ*+LPS-activated macrophages, they expose phosphatidylserine (PS) in their membrane that prevents the synthesis of NO. Also, parasite phagocytosis downregulates NOS2 expression and induces an anti-inflammatory response through the secretion of TGF-*β*, like what is observed when apoptotic cells are internalized. (b) During the acute phase of infection, there is an increase in depolarized mitochondria in CD4^+^ T cells and consequently mROS accumulation and PD-1 expression. In addition, ROS accumulation induces mitochondrial dysfunction, apoptosis, and poor T cell function, which can be reversed with antioxidants. The production of IFN-*γ*, and in consequence the classical activation of macrophages, diminishes with the elimination of T cells. Contrarily, parasite persistence is favored through the alternative activation of macrophages. (c) *T. cruzi* engages FasR, and T cell apoptosis is induced via the extrinsic pathway, leading to the activation of Caspases 8 and 3. Furthermore, the use of the Caspase 8 inhibitor zIETD or the pan-caspase inhibitor zVAD prevents T cell apoptosis in infected mice. (d) Treatment with zVAD during the acute phase of infection also results in decreased parasitemia and reduced apoptosis of splenocytes.

After parasite internalization, there is an important decrease in NOS2 expression and overexpression of TGF-*β* that could induce an anti-inflammatory response like what is observed during phagocytosis of apoptotic cells [117]. The role of NO in inducing apoptosis in vivo in cells infected with *T. cruzi* has been demonstrated in splenocytes from acutely infected BALB/c mice. These cells showed reduced viability and increased spontaneous apoptosis after 48 h in culture. However, cell viability was restored when NO production was inhibited by the addition of the L-arginine analog NG-monomethyl-L-arginine (L-NMMA) or monoclonal antibodies (mAbs) targeting IFN-*γ* or TNF-*α* [118] (Figure 2a). In addition to inhibiting NOS2 expression through PS exposure, *T. cruzi* also addresses the issue of NO production by directly affecting IFN-*γ*-producing cells. During the acute phase of *T. cruzi* infection, the parasite induces apoptosis and metabolic changes in the primary IFN-*γ*-producing cells of the adaptive immune response, namely, CD4^+^ and CD8^+^ T lymphocytes. This correlates with a reduction in proliferative responses upon T cell receptor (TCR) engagement [119, 120]. It has been shown that during the acute phase of CD, CD4^+^ T cells are characterized by an intensified metabolism, depolarized mitochondria, mROS accumulation, and expression of the PD-1 inhibitory receptor that may contribute to their poor function and enhanced apoptosis, which was reversed with antioxidants [121]. The induction of apoptosis of T cells impedes the production of IFN-*γ* and subsequently the classical activation of macrophages. Conversely, macrophages are activated to an M2 phenotype that favors parasite persistence [122] (Figure 2b). In vivo analyses have demonstrated that besides CD4^+^ T cells, apoptosis is present in other cells and organs during infection with *T. cruzi*, particularly in lymphoid organs [118, 123–128]. T cells from individuals with chronic cardiac CD and heart failure also undergo apoptosis and exhibit impaired proliferative capacity [129, 130], a phenomenon that has been observed in cardiac tissue from both experimental models and human patients [131, 132].

As can be seen, substantial evidence supports the fact that *T. cruzi* induces apoptosis of T and B cells. Nevertheless, the molecular mechanisms behind the induction of apoptosis by *T. cruzi* have not been completely deciphered. Indeed, elucidation of these mechanisms is of utmost importance as these molecules could serve as potential targets for restoring immune function during parasitic infections [133, 134]. In terms of the mechanisms employed by *T. cruzi* to trigger apoptosis, studies have demonstrated that T cells from *T. cruzi*-infected mice exhibit elevated expression of proapoptotic markers, including Fas (CD95), Fas ligand (FasL, CD95L), and activated Caspases 3 and 8 [135–139]. This presupposes the fact that the induction of apoptosis of T lymphocytes exerted by *T. cruzi* occurs through the extrinsic pathway by way of FasR with the consequent activation of Caspases 8 and 3 [136]. The role of FasL in the induction of apoptosis by *T. cruzi* has also been corroborated with the use of an antibody anti-FasL that blocked apoptosis induction in CD8^+^ T lymphocytes, increased T cell proliferation, improved the Th1 response that restored macrophage-mediated immunity to *T. cruzi* infection, and diminished peak parasitemia [135, 140] (Figure 2c). Similar effects have also been observed in T cells from infected FasL-deficient generalized lymphoproliferative disease (gld) mutant mice [135, 136]. In relation to the role of Caspase 8 in the induction of apoptosis by *T. cruzi*, it has been demonstrated that the Caspase 8 inhibitor zIETD blocks T cell apoptosis in infected mice, and the usage of the pan caspase inhibitor, zVAD, during acute infection reduces splenocyte apoptosis and parasitemia [137, 138] (Figure 2c). The pharmacological and genetic intervention towards the *T. cruzi* proapoptotic effect against effector T cells is desirable; nevertheless, unintended effects have been observed, such as the development of autoimmunity in models using FasL-deficient (gld) or Fas-deficient (lpr, lymphoproliferative) mice [141–143], as well as heightened cardiac inflammation in *T. cruzi*-infected mice treated with anti-FasL antibodies [144].

The role of FasR/FasL in the induction of apoptosis by *T. cruzi* has also been demonstrated in vivo. Patients in the chronic phase with CC symptoms show an increase in the serum level of FasL, which is indicative that apoptosis also occurs in this phase [129]. Furthermore, T cells from patients with CD experience defective proliferation due to FasL-Fas expression and activation-induced cell death [129, 130]. Interestingly, other participants of the extrinsic pathway do not seem to be involved since the use of antibodies against TNFR or TRAIL did not block the induction of apoptosis [135].

Other cells that have been shown to internalize *T. cruzi* trypomastigotes are neutrophils [145], and when they are infected with *T. cruzi* trypomastigotes of the Col1.7G2 (DTU I) or Y (DTU II), apoptosis is induced, which is an effective escape mechanism from the host immune response [146].

Additionally, the proapoptotic effect exerted by *T. cruzi* has also been observed in ex vivo experiments carried out with human placenta explants (HPEs). In the trophoblast, genic expression is regulated at the post-transcriptional level by micro-RNAs (miRNAs). miR-512-3p is an miRNA expressed in the placenta where it regulates differentiation and cell death through the suppression of c-FLIP (Caspase 8 inhibitor) [147]. The infection of HPE with *T. cruzi* trypomastigotes of the Y strain or the transfection of the explants with miR-512-3 increased both miRNA level as well as Caspases 3 and 8 activity and expression levels, determined by enzymatic assays and RT-qPCR, respectively. On the other hand, miR-512-3p inhibition prevented apoptotic cell death by decreasing Caspases 3 and 8 activity, as well as the expression of their mRNA's even in the presence of the parasite [148] (Figure 2d).

### 2.8. Apoptosis Inhibition by *T. cruzi*

**Figure 3:**
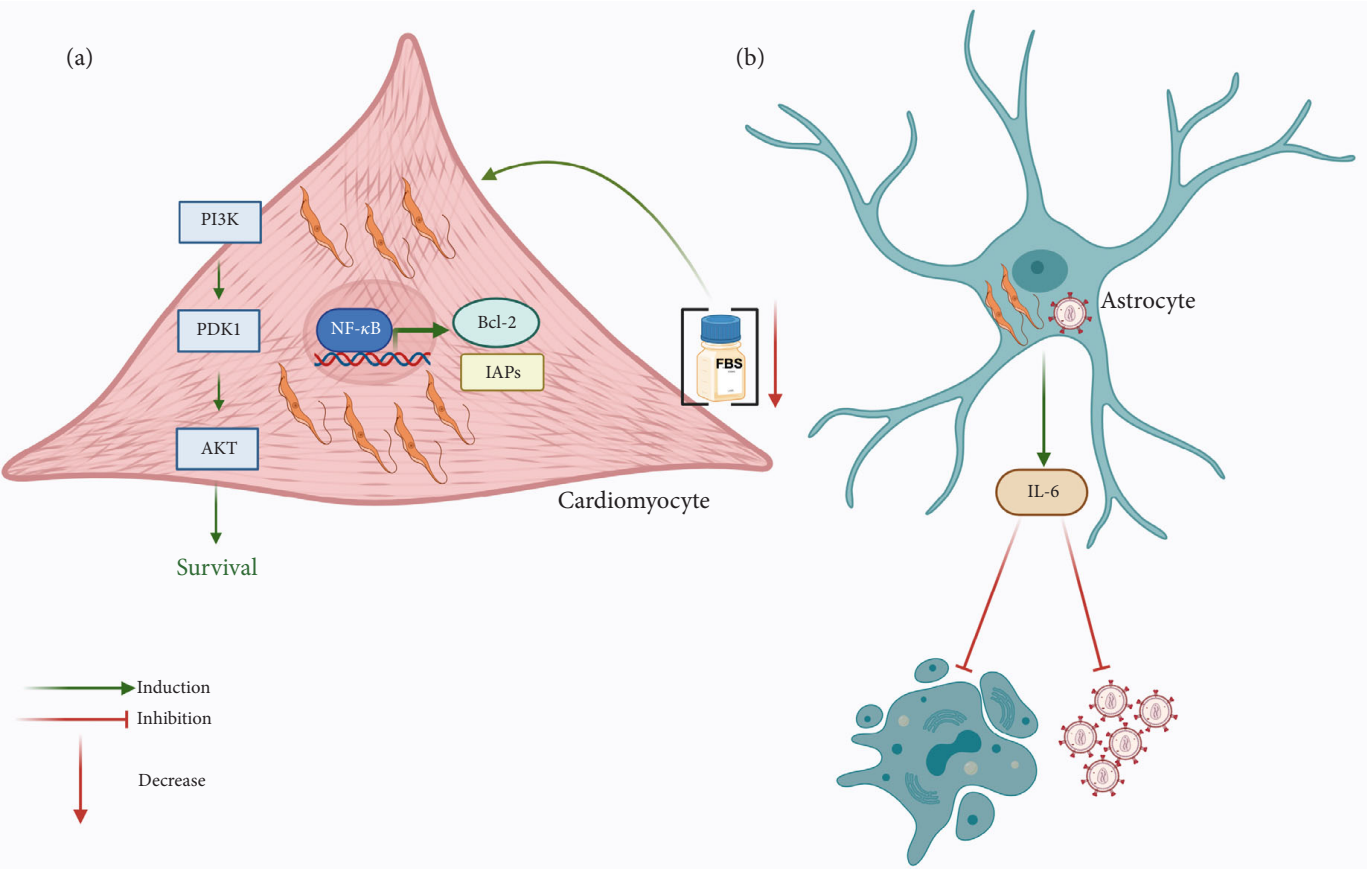
Inhibition of apoptosis by *Trypanosoma cruzi*. (a) *T. cruzi* enhances the survival of murine cardiomyocytes cultured under low-serum conditions. This effect is associated with increased Akt phosphorylation and upregulation of the antiapoptotic protein Bcl-2. Specifically, Bcl-2 expression has been linked to the activation of the transcription factor NF-*κ*B, which also regulates other antiapoptotic mechanisms, including the expression of inhibitors of apoptosis proteins (IAPs). (b) Coinfection of astrocytes with *T. cruzi* and HIV leads to the secretion of IL-6, which diminishes astrocyte apoptosis and viral replication.

As mentioned before, one of the most important cells in which *T. cruzi* induces apoptosis is IFN-*γ*-producing cells to block the ability of this cytokine to induce NO production. Contrarily, towards other cells, where the parasite needs to survive and replicate, the effect that has been observed is the inhibition of apoptosis. *T. cruzi* can infect many nucleated cells but has a special tropism towards macrophages, muscle, and nervous cells. One very interesting host cell is the cardiomyocyte, where *T. cruzi* can survive for years. The infection with *T. cruzi* of murine cardiomyocytes cultured in medium with minimal serum concentration showed an increased survival of the cells, which correlated with a rise in Akt phosphorylation and in the expression of Bcl-2 [149]. In particular, the expression of the antiapoptotic protein Bcl-2 has been linked to the transcription factor NF-*κ*B along with other antiapoptotic responses such as IAPs [150, 151]. In the case of the infection with *T. cruzi*, it has been shown that NF-*κ*B participates in the protection of apoptosis of neonatal rat cardiomyocytes and H9c2 (rat cardiomyoblast cell line) cells [152]. It seems that prevention of cardiac cell apoptosis due to the engagement of NF-*κ*B during *T. cruzi* infection is dependent on the parasite since soluble factors do not exert any effect and lead to the production of antiapoptotic molecules and the inhibition of Caspase 3 activation [152] (Figure 3).

Relative to the PI3K/Akt signaling pathway that has a preponderant role in cellular survival and, thus, promotes downregulation of apoptosis, it is also an important objective of pathogens to modulate this type of cell death. Thus, it is not surprising that *T. cruzi* targets this survival pathway to inhibit cardiomyocyte apoptosis [149]. On the other hand, Bcl-2 is an antiapoptotic protein whose overexpression is anticipated as a mechanism employed by *T. cruzi* to inhibit apoptosis, as has been demonstrated during the infection of cardiomyocytes and H9c2 cells [149, 151] (Figure 3a).

In addition to the preferential tropism of *T. cruzi* towards macrophages and cardiomyocytes, it can also infect nervous cells, such as Schwann cells and astrocytes. It has been shown that the coinfection of astrocytes with *T. cruzi* and HIV favors the interaction between both pathogens that diminishes HIV replication and astrocyte apoptosis by cellular (IL-6) and parasite-released soluble factors [153] (Figure 3b).

### 2.9. *T. cruzi* Molecules That Modulate Apoptosis

**Figure 4:**
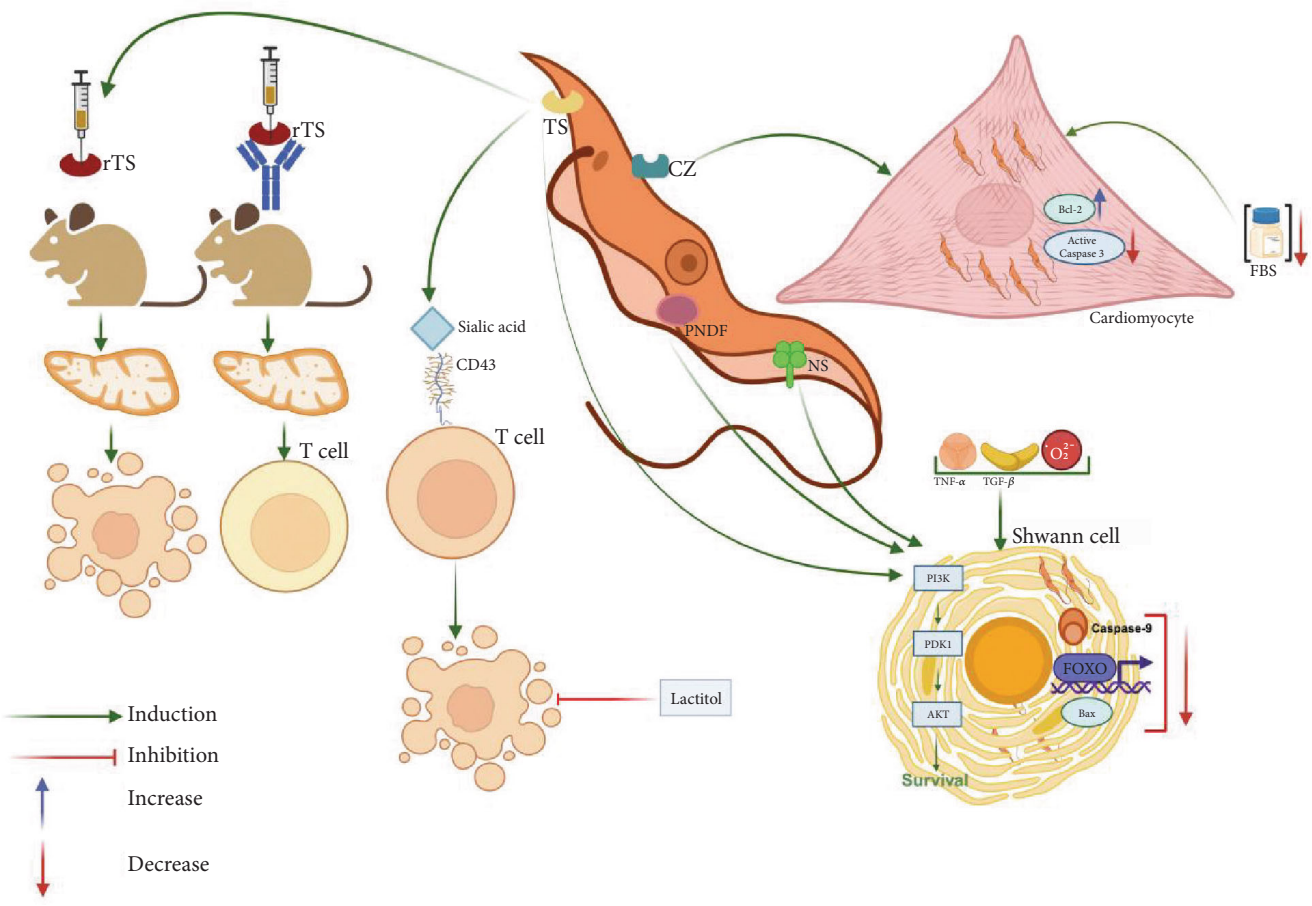
*T. cruzi* molecules that modulate apoptosis. TS is an enzyme localized on the surface of *T. cruzi* or secreted far from the site of infection. It transfers sialic acid from the glycoconjugates present in mammalian cells to the parasite surface and has the capacity to trigger apoptosis. When recombinant TS is administered to mice, it induces apoptosis in the thymus, which is prevented with neutralizing anti-TS antibodies. Apoptosis is also observed in T lymphocytes and monocytes through the sialylation of the mucin CD43, which is prevented with lactitol, a transferase inhibitor. Cruzipain is another *T. cruzi* surface molecule that favors parasite survival. It has been shown that in cardiomyocytes cultured under stress conditions with minimal serum concentration, there is an increase in Akt phosphorylation and the expression of Bcl-2. Additionally, TS and other parasite molecules, such as a neuraminidase and a parasite-derived neurotrophic factor (PNDF), promote parasite survival in infected Schwann cells despite proapoptotic stimuli induced by TNF-*α*, TGF-*β*, or H_2_O_2_. Particularly, TS interacts with Akt, which increases its expression and inhibits the expression of three proapoptotic molecules: Bax, Caspase 9, and the transcription factor FOXO, which altogether promote cell viability.

In addition to the ability of *T. cruzi* to induce apoptosis, it has been shown that the effect can also be exerted by some molecules present in the parasite. This is the case of the *T. cruzi* TS, an enzyme that during infections is localized on the parasite surface or secreted far from the site of infection [154]. TS transfers sialic acid from mammalian cell glycoconjugates to the parasite surface. Interestingly, TS can trigger apoptosis in the thymus, as has been demonstrated in mice administered with recombinant TS. Contrarily, mice treated with a neutralizing anti-TS antibody did not show abnormalities in the organ [155, 156]. These important findings were later corroborated by the same research group using the TUNEL technique and showed that the sialylation of the mucin CD43, constitutively expressed on the surface of T lymphocytes and monocytes, induced apoptosis [156] (Figure 4). Interestingly, this effect was absent in mice treated with lactitol, an inhibitor of the transferase enzyme [156]. Beyond TS, other factors derived from both the parasite and the host have been implicated in thymocyte death during acute *T. cruzi* infection. These include Gal-3, extracellular ATP, glucocorticoids, and androgens [127, 155, 157–162]. Notably, neither Fas—known for its role in T cell apoptosis—nor perforin appears to contribute to the thymic atrophy induced by *T. cruzi* [163].

Regarding glucocorticoids, their involvement in in vivo thymocyte apoptosis during infection has been linked to the activation of Caspases 8, 9, and 3. Gal-3 also plays a role in the modulation of apoptosis by *T. cruzi*. In humans, Gal-3 is encoded by the LGALS3 gene, has a molecular weight of approximately 31 kDa, and features a carbohydrate-recognition domain (CRD) with high affinity for glycoconjugates, particularly *β*-galactosides [164]. During *T. cruzi* infection, the parasite has been shown to differentially regulate Gal-3-dependent survival pathways. In HeLa cells, *T. cruzi* infection inhibits apoptosis, as evidenced by distinct characteristics in Gal-3-deficient HeLa cells, including loss of mitochondrial membrane potential, increased Caspase 3 activity, and enhanced proteolytic cleavage of PARP [165]. Contrarily, in the thymus, it is involved in thymocyte death [162].

Another important molecule involved in the modulation of apoptosis is cruzipain, which is a major multifunctional cysteine peptidase that participates in several physiological and pathological processes critical for a successful parasitic infection [166]. As already mentioned, cardiomyocytes are a major host cell for *T. cruzi*, and the inhibition of apoptosis in these cells has been demonstrated. It was shown that cruzipain also favored parasite survival when cardiomyocytes were cultured in media with minimal serum concentration, and the phenomenon was also associated with an increase in Akt phosphorylation and the expression of Bcl-2 [149]. Interestingly, the cultures treated with cruzipain showed lesser Caspase 3 activation despite serum deprivation, which suggests that cruzipain may be responsible for the antiapoptotic effect [149] (Figure 4).

Regarding the infection of nervous cells by *T. cruzi*, Chuenkova and Pereira Perrin observed that Schwann cells infected with *T. cruzi* trypomastigotes can survive despite proapoptotic stimuli induced by TNF-*α*, TGF-*β*, or H_2_O_2_. The effect was associated with the interaction of some parasite molecules, particularly TS, a neuraminidase, and a parasite-derived neurotrophic factor (PNDF), with Akt, which increases its expression and inhibits the expression of three proapoptotic molecules: Bax, Caspase 9, and the transcription factor FOXO, which altogether promoted cell viability [167] (Figure 4).

### 2.10. Apoptosis-Like Death in *T. cruzi*

Not long after apoptosis was identified in metazoans, different studies were carried out in unicellular organisms to find out if similar processes existed. Docampo et al. performed studies with *T. cruzi* by treating epimastigotes with DNA synthesis inhibitors such as *β*-lapachone or o-naftoquinone. Interestingly, through transmission electron microscopy, they identified nuclear and cytoplasmic characteristics indicative of apoptosis, including cell membrane blebbing, chromatin condensation, and mitochondrial membrane modifications [168]. These interesting findings lead other research groups to deepen in this type of cell death in *T. cruzi* and found other apoptotic features such as DNA fragmentation, PS externalization, loss of mitochondrial membrane potential, and cytochrome C release [169, 170]. Although these features resemble metazoan apoptosis, many molecules involved in the process present notable differences, which is why the phenomenon observed in *T. cruzi* has been named “apoptosis-like cell death” [171].

As previously said, one of the molecules that play a leading role in apoptosis is caspases, and these enzymes are not present in *T. cruzi.* Nevertheless, in the genome of *T. cruzi*, there are two genes, TcMCA3 and TcMCA5, that encode metacaspases, which are cysteine proteases structurally like caspases but, unlike them, exhibit specificity for basic amino acids and require millimolar concentrations of calcium for activity [169, 170]. It has been shown that the different parasite stages (epimastigotes, MTs, blood trypomastigotes, and amastigotes) express TcMCA3, while TcMCA5 is only present in epimastigotes [172]. Interestingly, data obtained with immunofluorescence microscopy showed that both metacaspases change their subcellular localization and translocate to the nucleus when programmed cell death is induced in the parasites by treatment with human serum [172]. Additionally, it has been observed that epimastigotes are more prone to programmed cell death with an increase in TcMCA5 expression [172]. Contrarily, TcMCA3 safeguards epimastigotes against natural cell death and appears to play a significant role in their differentiation into infectious MTs [173].

Another molecule that may play a role in apoptosis-like cell death in *T. cruzi* is elongation factor 1 (EF-1). This factor, typically found in the nucleus and cytoplasm of eukaryotic cells, consists of two subunits (EF-1*α* and EF-*βγδ*) and is significantly involved in protein synthesis, as well as in processes like mitosis and cell proliferation [174]. Interestingly, by employing FITC-labeled antibodies against TcEF-1*α*, it was demonstrated that this molecule accumulates in the nucleus of *T. cruzi* epimastigotes cultured for over 13 days, which exhibited apoptotic characteristics [175]. Research performed later showed that changes in the expression level of EF-1*α* modify the apoptosis rate in a murine erythroleukemia cell line [176]; thus, TcEF-1*α* could be considered an apoptosis marker in *T. cruzi*.

## 3. Conclusions

The survival for years of *T. cruzi* in its host cells has been an intriguing matter. Great efforts have been made to elucidate the molecular basis underlying *T. cruzi* survival in its host. During its evolution, this parasite has developed multiple strategies that have permitted it to persist in hostile environments. By differentially regulating apoptosis, *T. cruzi* seems to have devised a strategy to evade the immune system while preserving the survival of its preferred host cell type. This mechanism could contribute to its persistence in cardiac muscle, facilitate transmission to other hosts, and potentially lead to the development of chronic cardiac CD.

The effective balance between parasite persistence, immune regulation, and cardiovascular damage may be closely linked to the differential modulation of host apoptotic functions, either directly or indirectly, following *T. cruzi* infection. Gaining a comprehensive understanding of the biochemical and molecular interactions between the parasite and its host is vital for identifying novel molecular targets, which could be studied in the future to develop strategies to disrupt the progression of CD. The signaling pathways responsible for mediating these interactions remain to be fully elucidated.
